# Theoretical basis validation and oxidative stress markers for cancer prevention clinical trials of aspirin

**DOI:** 10.1038/s41598-023-49254-3

**Published:** 2023-12-11

**Authors:** Takahiro Hamoya, Susumu Tomono, Shingo Miyamoto, Gen Fujii, Keiji Wakabayashi, Michihiro Mutoh

**Affiliations:** 1 Department of Molecular-Targeting Prevention, Kyoto Prefectural University of Medicine, Kyoto, Japan; 2 Department of Microbiology and Immunology, Aichi Medical University, Nagakute, Aichi 480-1195 Japan; 3 Graduate Division of Nutritional and Environmental Sciences, University of Shizuoka, Shizuoka, 422-8526 Japan; 4 Epidemiology and Prevention Division, Center for Public Health Sciences, National Cancer Center, Tokyo, 104-0045 Japan

**Keywords:** Cancer prevention, Tumour biomarkers

## Abstract

Aspirin, a nonsteroidal anti-inflammatory drug, has been proven effective in a clinical trial of carcinogenesis blockade. However, various modes of action have been reported for these effects. Thus, in this study, we aimed to present reasonable mode of actions as a proof of concept for human trials, especially trials for patients with familial adenomatous polyposis (FAP). Aspirin treatment at 1000 ppm inhibited intestinal tumorigenesis in FAP model Min mice. As a mode of action, aspirin regulated β-catenin signaling, inflammation, and oxidative stress both in vivo and in vitro. Furthermore, we examined novel markers predictive of aspirin treatment based on liquid biopsy. Here, we demonstrated that aspirin reduced the levels of reactive carbonyl species in the serum of Min mice. These data are expected to be of use for proof of concept of aspirin human trials and implied for the prediction of aspirin efficacy.

## Introduction

Patients with familial adenomatous polyposis (FAP) are a high-risk group for colorectal cancer (CRC) due to a mutation in adenomatous polyposis coli (*APC)*, a tumor suppressor gene, which causes the development of many intestinal polyps (mainly, adenoma as a precancerous lesion). It is assumed that treatment at the precancerous lesion stage can strongly prevent cancer development. Recently, we reported for the first time, to the best of our knowledge, that low-dose aspirin (100 mg/day) for 8 months significantly inhibited the recurrence/growth of intestinal polyps as the primary endpoint in patients with FAP without colorectomy (J-FAPP Study IV)^1^.

CRC was the second leading cause of cancer deaths (about 935,000 deaths) worldwide in 2020^2^. The global burden of CRC is expected to increase by 60% to more than 3.2 million new cases and 1.6 million deaths in 2040^3^. In recent years, the increase in the number of younger patients with CRC under the age of 50 has become a serious health issue in the United States^4,5^. Sporadic CRC is a worldwide problem, and the mechanism of occurrence is often explained by the adenoma-carcinoma sequence such that cancer prevention strategies for patients with FAP can be directly applied to sporadic CRC as well.

Aspirin has been investigated as a cancer chemoprevention agent against CRC^6,7^. For a long time, it has been reported epidemiologically that aspirin suppresses CRC^8^, and clinical trials in many countries have reported that aspirin reduces the risk of CRC^6,7^. Aspirin is a non-steroidal anti-inflammatory drug (NSAID) and is known as a cyclooxygenase (COX) inhibitor, but the mechanism of inhibiting carcinogenesis by aspirin has not yet been clarified. Its preventive mechanisms have been approached through two pathways, the COX-pathway and the non-COX-pathway^7^. In in vitro settings, many reports suggested aspirin affects cell proliferation, inflammation, and apoptosis regardless of prostaglandin production (non-COX-pathway)^7,9^. However, the polyp inhibitory effect of aspirin in vivo has long been controversial^10^. Therefore, we used *Apc*‐mutant mice (Min mice), a model of FAP, in which many intestinal polyps were developed through activation of β‐catenin signaling, to evaluate the effects of aspirin.

Min mice are a well-known FAP model mice with mutations in the *Apc* gene, which is the causative gene of FAP^11^. Several recent studies have reported that aspirin inhibited the number of intestinal polyps in Min mice that were induced to develop inflammatory colon carcinogenesis using azoxymethane/dextran sulfate sodium (AOM/DSS) treatment^12,13^. However, because of the limited number of studies showing the polyp inhibitory effect of aspirin against hereditary intestinal polyp formation, clinical biomarker studies to assess the effects of aspirin have also been few in number.

This study aimed to present the mode of action for the proof of concept (POC) of a human trial, the J-FAPP Study IV. The modes of action of aspirin will be anti-inflammation, improvement of abnormal β-catenin signaling, and anti-oxidative stress. To this end, in vitro and in vivo experiments showed that aspirin regulated three states (cell proliferation, inflammation, and oxidative stress) related to colon carcinogenesis and decreased intestinal tumorigenesis in Min mice. Furthermore, we used this model and searched for novel markers predictive of cancer prevention based on liquid biopsy.

## Results

### Dose-dependent effects of aspirin on transcriptional activity and mRNA expression levels

**Figure 1:**
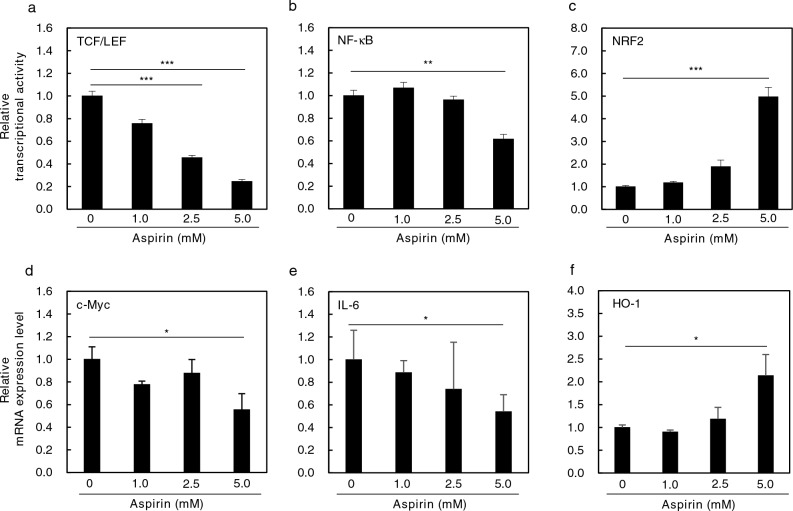
Effects of aspirin on TCF/LEF, NF-κB, and NRF2 promoter transcriptional activity and downstream target mRNA levels in HCT116 cells. (**a**)–(**c**) HCT116-TCF/LEF-Luc cells (**a**), HCT116-NF‐κB-Luc cells (**b**), and HCT116-NRF2-Luc cells (**c**) were treated with the indicated dose of aspirin for 24 h, and luciferase activities were measured using a luminometer. The basal luciferase activity level is set to 1.0. (**d**)–(**f**) HCT116 cells were seeded in 12-well plates (4 × 10^5^ cells per well) and cultured in medium containing 1.0, 2.5, and 5.0 mM aspirin for 24 h. After 24 h, c-Myc mRNA (**d**), IL-6 mRNA (**e**), and HO-1 mRNA (**f**) levels were evaluated in HCT116 cells by quantitative real-time PCR analysis. The basal mRNA levels of the control were set to 1.0. Data were normalized to GAPDH mRNA levels. Data presented are the mean ± SD (n = 4). Student’s *t* test, *p < 0.05, **p < 0.01, and ***p < 0.001 compared with 0 mM aspirin.

Three states (cell proliferation, inflammation, and oxidative stress) are known to be involved in the early and accelerated stages of colon carcinogenesis. To examine the effects of aspirin on specific promoter transcriptional activity in the three states, stably transfected HCT116-T cell factor/lymphoid enhancer factor (TCF/LEF)-Luc, HCT116-nuclear factor-κB (NF‐κB)-Luc, and HCT116-NF-E2-related factor 2 (NRF2)-Luc cells were treated with 1.0, 2.5, and 5.0 mM aspirin for 24 h (Fig. 1). In HCT116-TCF/LEF-Luc cells, 2.5 and 5.0 mM aspirin treatment decreased TCF/LEF promoter transcriptional activity by 54.6% (*p* < 0.001) and 75.6% (*p* < 0.001), respectively, compared with the untreated control (Fig. 1a). In HCT116-NF-κB-Luc cells, 5.0 mM aspirin treatment decreased NF-κB promoter transcriptional activity by 38.5% (*p* < 0.01) compared with the untreated control (Fig. 1b). In HCT116-NRF2-Luc cells, 5.0 mM aspirin treatment increased NRF2 promoter transcriptional activity by 4.96-fold (*p* < 0.001) compared with the untreated control (Fig. 1c). Similar results were obtained in the DLD-1-TCF/LEF-Luc, DLD-1-NF-κB-Luc and DLD-1-NRF2-Luc cells (Supplementary Fig. 1a–c).

In order to clarify the effects of aspirin on the downstream targets of TCF/LEF, NF-κB, and NRF2, mRNA expression regulated by each signaling pathway was evaluated in HCT116 cells. Treatment with 5 mM aspirin for 24 h in HCT116 cells reduced c-Myc mRNA levels by 44.6% and interleukin (IL)-6 mRNA levels by 46.0% compared with the untreated control (Fig. 1d,e). In addition, treatment with 5 mM aspirin for 24 h in HCT116 cells increased heme oxygenase (HO)-1 mRNA levels by 2.14-fold (*p* < 0.01) (Fig. 1f).

### Inhibition of small intestinal polyp formation in Min mice by aspirin

We next tried to determine whether the effects of aspirin on TCF/LEF, NF-κB, and NRF2 signal regulation in vitro could also be seen in vivo. Aspirin was administered to *Apc*-mutant multiple intestinal neoplasia (Min) mice that develop several intestinal polyps due to *Apc* deletion. Administration of 1000 ppm (= 5.55 mM) aspirin for 8 weeks did not affect body weight, food intake, or clinical symptoms throughout the experimental period. There was no difference in average daily food intake among Min mice in the sterile water group (n = 9 mice) or aspirin‐treated group (n = 10 mice). In addition, no changes in organ weight, which was measured as an indicator of toxicity, were observed. Furthermore, gastrointestinal mucosa hemorrhage was not detected through macroscopic observations.

**Figure 2:**
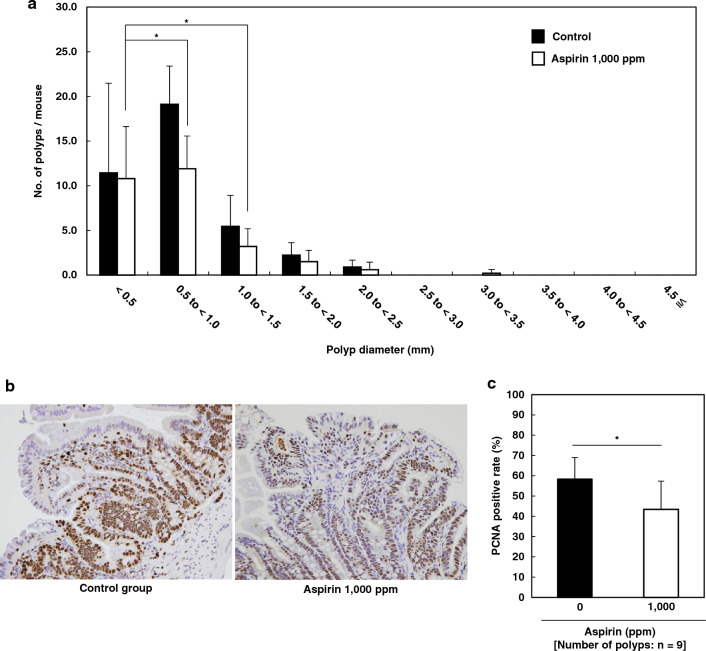
Effects of aspirin on the size distribution and proliferating cell nuclear antigen (PCNA)‐positive cells of intestinal polyps. (**a**) Min mice were allowed to drink sterile water (black column) or a sterile water containing 1000 ppm aspirin (open column) for 8 weeks. The number of polyps per mouse in each size class is given as the mean ± SD. *p < 0.05 versus the control group. (**b**) and (**c**) Representative data of PCNA immunohistochemical staining from each group (left, sterile water group; right, aspirin-treated group) are shown. Percentages of PCNA‐positive cells were calculated as shown (**c**). The number of examined polyps is shown in parentheses. Data are presented as mean ± SD. *p < 0.05 versus control group.

Table 1 summarizes the data regarding the number and distribution of intestinal polyps in the sterile water group and aspirin-treated group. The majority of polyps developed in the small intestine, whereas only a few developed in the colorectum. Aspirin treatment decreased the total number of polyps to 72.1% of that of the untreated control (*p* < 0.05 vs. control group). The number of polyps in the distal segment of the small intestine was 68.7% of that in the untreated control (*p* < 0.05 vs. control group). No significant differences in the numbers of polyps were observed in other segments of the small intestine or colorectum. Figure 2a shows the size distributions of the intestinal polyps in the sterile water and aspirin-treated groups. The majority of polyps were less than 3 mm in diameter. Treatment with 1000 ppm aspirin significantly reduced the size of polyps to between 0.5 and 1.5 mm (*p* < 0.05 vs. control group).

**Table 1:** Number of intestinal polyps/mouse in Min mice.

Aspirin (ppm)	No. of mice	Small intestine			Colon	Total
		Proximal	Middle	Distal		
0	9	6.0 ± 3.1	11.7 ± 4.7	21.4 ± 6.9	0.0 ± 0.0	39.1 ± 11.6
1000	10	5.9 ± 1.7	7.4 ± 4.8	14.7 ± 4.8*	0.2 ± 0.4	28.2 ± 5.5*

Date are mean ± SD.

Significantly different from the control untreated group at *p < 0.05.

Immunohistochemical staining of proliferating cell nuclear antigen (PCNA), which represents cell growth, supported the effect of aspirin on the growth of tumor epithelial cells. PCNA‐positive cells occupied 58.4% of the tumor epithelial cells in polyps of the sterile water group and 43.4% in the aspirin‐treated group (Fig. 2b,c).

### Improvement in cell proliferation-, inflammation-, and oxidative stress-related mRNA expression in small intestinal polyps of Min mice treated with aspirin

**Figure 3:**
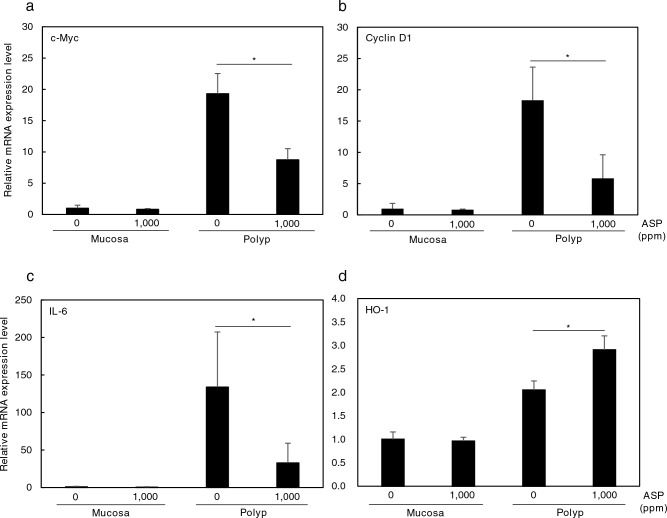
mRNA expression levels of cell proliferation-, inflammation-, and oxidative stress-related factors in the small intestine of Min mice. (**a**)–(**d**) Quantitative real-time PCR analysis was performed to evaluate c-Myc mRNA (**a**), Cyclin D1 mRNA (**b**), IL-6 mRNA (**c**), and HO-1 mRNA (**d**) expression levels in the aspirin-treated group the samples obtained from in the non-polyp (mucosa) and polyp-containing parts of the intestines of Min mice. Each expression level in the non-polyp (mucosa) parts of the intestines in the control group (0 ppm) was set as 1.0. Data were normalized using GAPDH mRNA levels. Data are the mean ± SD (n = 4). Student’s *t* test, *p < 0.05 versus control group.

To clarify the mechanisms underlying aspirin-induced inhibition of small intestinal polyp formation, cell proliferation-, inflammation-, and oxidative stress-related gene expression in the non-polyp (mucosa) parts and polyp-containing parts of the small intestine were investigated (Fig. 3). Real-time PCR revealed that the 1000 ppm aspirin significantly suppressed the mRNA expression levels of c-Myc and Cyclin D1, which are cell proliferation-related genes, in small intestinal polyps by 64.4% (*p* < 0.05) and 56.8% (*p* < 0.05), respectively, of that in the untreated control (Fig. 3a,b). Real-time PCR revealed that polyps had 100-fold higher mRNA expression levels of IL-6, which is an Inflammation-related gene, than non-polyp (mucosa) parts. In addition, in the 1000 ppm aspirin-treated group mRNA expression levels of IL-6 were significantly suppressed in the small intestinal polyp-containing part by 75.4% (*p* < 0.01) compared with that in the untreated control (Fig. 3c). Real-time PCR revealed that the 1000 ppm aspirin significantly increased mRNA expression levels of the oxidative stress-related gene HO-1 by 1.41-fold (*p* < 0.05) in small intestinal polyps compared with that in the untreated control (Fig. 3d). In the non-polyp (mucosa) parts, however, no differences in c-Myc, Cyclin D1, IL-6, or HO-1 mRNA expression levels between the aspirin-treated and the untreated group were observed (Fig. 3a–d).

### Decreased levels of oxidative stress-related markers in the serum of Min mice treated with aspirin

**Figure 4:**
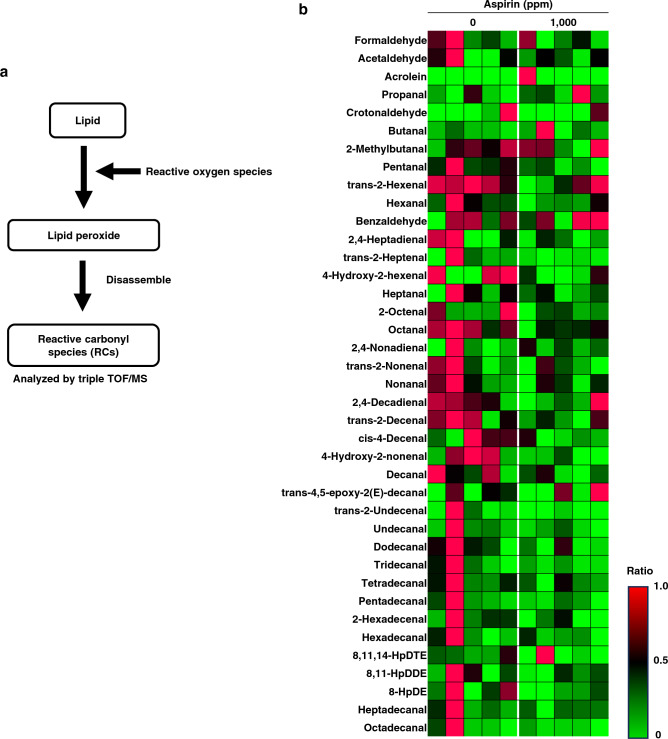
The detection levels of RCs in the serum of Min mice treated with aspirin. (**a**) Pathway diagram of RC production in vivo. (**b**) Heat map showing the amount of RCs detected in the serum of Min mice in the control group versus aspirin-treated group. Green color indicates lower than mean intensity, and red represents higher than mean intensity.

In this study, we found that aspirin treatment inhibited the development of the number of small intestinal polyps (Fig. 2) and activated oxidative-related gene expression levels at polyp-containing sites in Min mice (Fig. 3d). Thus, in the next experiment we aimed to search for index markers for the suppressive effects of aspirin among oxidative stress markers. We analyzed reactive carbonyl species (RCs) as a method for detecting oxidative stress markers. RCs are degradation products resulting from the transformation of lipids into oxidized lipids by reactive oxygen species in vivo (Fig. 4a). Based on a liquid biopsy, we searched for markers in serum. We listed the levels of 39 well-known small molecular weight RCs in the serum. The samples obtained from Min mice treated with or without aspirin were analyzed using liquid chromatography/mass spectrometry (LC/MS). As a result, the levels of pentanal, 4-hydroxy-2-hexenal (4-HHE), octenal, 4-hydroxy-2-nonenal (4-HNE), trans-2-undecenal, and 2-hexadecenal were significantly lower in the aspirin-treated group compared with that in the untreated control group (Fig. 4b; Table 2).

**Table 2:** The levels of RCs detected in the serum (pmol/ml).

Compounds	Aspirin (ppm)	
	0	1000
Formaldehyde	3239.1 ± 1068.2	2732.5
Acetaldehyde	7651.4 ± 1842.6	7219.0 ± 866.3
Acrolein	n.d	3.7 ± 8.2
Propanal	188.1 ± 41.2	223.0 ± 59.6
Crotonaldehyde	12.3 ± 16.9	6.2 ± 13.9
Butanal	91.8 ± 57.9	202.1 ± 250.2
2-Methylbutanal	10.3 ± 5.7	10.2 ± 8.1
Pentanal	52.8 ± 23.8	20.0 ± 14.0*
Trans-2-Hexenal	2.8 ± 0.2	2.2 ± 0.6
Hexanal	182.1 ± 69.8	116.1 ± 46.4
Benzaldehyde	31.0 ± 14.9	34.2 ± 16.6
2,4-Heptadienal	3.9 ± 3.7	3.2 ± 1.9
Trans-2-Heptenal	2.9 ± 3.2	0.7 ± 0.3
4-Hydroxy-2-hexenal	15.9 ± 14.5	4.1 ± 6.1*
Heptanal	30.6 ± 12.5	25.3 ± 5.4
2-Octenal	0.5 ± 0.7	n.d
Octanal	44.5 ± 7.7	30.7 ± 7.3*
2,4-Nonadienal	6.2 ± 5.7	9.8 ± 0.6
Trans-2-Nonenal	7.3 ± 2.4	5.8 ± 1.6
Nonanal	547.2 ± 200.5	426.0 ± 141.4
2,4-Decadienal	0.8 ± 0.5	0.3 ± 0.7
Trans-2-Decenal	1.1 ± 0.7	0.3 ± 0.4
Cis-4-Decenal	3.8 ± 0.8	3.1 ± 0.5
4-Hydroxy-2-nonenal	10.8 ± 6.7	3.0 ± 2.4*
Decanal	96.9 ± 27.5	74.9 ± 16.5
Trans-4,5-epoxy-2(E)-decanal	8.7 ± 8.3	9.5 ± 12.3
Trans-2-Undecenal	6.0 ± 0.8	2.2 ± 3.1*
Undecanal	37.4 ± 13.7	29.7 ± 4.7
Dodecanal	57.6 ± 17.4	44.4 ± 11.9
Tridecanal	1479.6 ± 625.0	1071.2 ± 122.7
Tetradecanal	65.4 ± 25.6	47.3 ± 15.7
Pentadecanal	4613.2 ± 1833.1	3500.2 ± 558.2
2-Hexadecenal	5.9 ± 2.2	2.1 ± 2.9*
Hexadecanal	112.3 ± 44.4	93.5 ± 18.1
8,11,14-HpDTE	6.1 ± 0.9	3.0 ± 4.5
8,11-HpDDE	5.1 ± 4.0	3.4 ± 2.3
8-HpDE	7.2 ± 5.3	2.9 ± 2.7
Heptadecanal	124.6 ± 64.7	93.7 ± 24.5
Octadecanal	1085.7 ± 686.5	691.9 ± 116.7

Date are mean ± SD (n = 5).

Significantly different from the control untreated group at *p < 0.05.

## Discussion

In this study, aspirin was shown to affect TCF/LEF, NF-κB, and NRF2 promoter transcriptional activities and downstream factors (c-Myc, IL-6, and HO-1) in human colon cancer cells. Moreover, we showed a significant reduction in the number of small intestinal polyps associated with aspirin treatment in drinking water. Furthermore, in the aspirin-treated group, the expression levels of cell proliferation (c-Myc and Cyclin D1)-, inflammation (IL-6)-, and oxidative stress (HO-1)-related factors in small intestinal polyps were strongly affected. In addition, the aspirin-treated group had reduced levels of pentanal, octenal, trans-2-undecenal, 2-hexadecenal, 4-HHE, and 4-HNE, in the serum of Min mice. Our data indicate that aspirin modulates three states (cell proliferation, inflammation, and oxidative stress) involved in the early and accelerated stages of colon carcinogenesis, and as a result, the candidate of novel serum predictive markers were obtained.

In vitro experiments showed that aspirin has anti-tumor effects via the non-COX-pathway, such as decreased transcriptional activity of TCF/LEF and c-Myc (suppression of cell proliferation) and increased transcriptional activity of NRF2 and HO-1 (enhancement of oxidative stress). TCF/LEF transcriptional activity is mainly regulated by the Wnt signaling pathway, and most colon cancer cells have an activated state of Wnt signaling^14^. It has been reported that NSAIDs degrade β-catenin in cells and inhibit the Wnt/β-catenin pathway^15^. NRF2 transcriptional activity is also a factor involved in the defense system against oxidative stress conditions in cells^16^. Aspirin has free radical scavenging properties and is a potent antioxidant in many oxidative stress‐relevant diseases^17^. The results of these studies support the suppression of TCF/LEF transcriptional activity and increase in NRF2 transcriptional activity by aspirin treatment. In addition, it has been reported that Wnt signaling and oxidative stress are closely related to inflammation^18^. Thus, the suppression of NF-κB transcriptional activity and IL-6 mRNA expression levels, which are thought to be in the COX-pathway, may contribute to the suppression mechanism of CRC. In the future, it will be necessary to examine in detail the relationship between each factor with aspirin treatment.

In vivo experiments, regarding the inhibition of small intestinal polyp formation by aspirin, three possible mechanisms were suggested from previous studies^19,20^. One is inhibition of Wnt signaling by aspirin, the second is based on the anti-inflammatory function of aspirin, and the third is lowering of oxidative stress by aspirin. In this study, the aspirin-treated group suppressed c-Myc and Cyclin D1 mRNA levels in small intestinal polyps in Min mice. Both c-Myc and Cyclin D1 are cell proliferation-related factors that are induced by activation of Wnt signaling through *APC* gene mutations found in ~ 80% of CRC^21,22^. So far, it has been suggested that suppression of excessive Wnt signaling is involved in polyp inhibition experiments using Min mice^23–25^. Previous studies reported that aspirin suppressed the expression levels of c-Myc and Cyclin D1 in many cancer cells and also suppressed cell proliferation-related marker proteins, such as Ki67 and PCNA^26–28^. Suppression of c-Myc and Cyclin D1 observed in the aspirin-treated group may have contributed to inhibition of the growth of polyp size especially in the case of small polyps.

The second is based on the anti-inflammatory function of aspirin. It is well-known that chronic inflammation is a cancer risk^29^. IL-6 is also a central molecule that promotes inflammation, and one of the factors that plays an important role in the growth of colorectal adenomas^30^. In this study, aspirin suppressed the mRNA expression levels of IL-6 in the small intestinal polyp region in Min mice. IL-6 is regulated by inflammation-related transcriptional factors, such as nuclear factor kappa B and activator protein-1, and it is reported that aspirin suppresses these transcriptional activities^31,32^. Furthermore, previous studies have suggested that suppression of IL-6 expression is involved in the reduction in small intestinal polyp formation in Min mice^33–36^.

The third is regarding the suppression of oxidative stress. To search for the mechanism and for use as a future biomarker, we comprehensively examined oxidative stress markers in this study. To this end, we measured RCs, oxidative stress markers, in serum^37–39^. Searching for predictive markers of aspirin efficacy is important because aspirin has been reported to suppress the recurrence of intestinal polyps in human clinical trials, and it is expected that aspirin will soon be used clinically as a CRC preventive agent^1,40,41^.

Aspirin treatment resulted in a decrease in serum 4-HHE and 4-HNE in Min mice. 4-HHE and 4-HNE are typical markers of oxidative stress, and they are increased in patients with lung cancer and colon cancer^42,43^. Of note, 4-HNE is known as an endogenous carcinogen^44,45^. It has been reported that knock-down of glutathione S-transferase alpha 4, which is a 4-HNE detoxifying enzyme, in Min mice increased the multiplicity of colon adenoma and decreased the survival rate^46,47^. In the present study, the levels of pentanal and octenal, which are members of the volatile organic compounds^48^, were also decreased in the serum of Min mice in the aspirin-treated group. Volatile organic compounds may be relevant as new cancer biomarkers for early stage cancer because their expression is increased in patients with early stage lung cancer and colon cancer^48,49^. In the future, it will be necessary to confirm the usefulness of each marker or a combination of several markers in human clinical trials.

In conclusion, we demonstrated a significant reduction in the number of small intestinal polyps following aspirin treatment. Furthermore, the RCs examined in this study are expected to be of use for evaluating the efficacy of aspirin in future human trials after accumulating more evidences. And, we consider that both COX-pathway and non-COX pathway are important for establishing POC in clinical trials of aspirin. Of note, our study presents the basis for the mode of action for the POC in the human trial, J-FAPP Study IV.

## Material and methods

### Chemicals

Aspirin was purchased from SIGMA-Aldrich (St. Louis, MO, USA). Acrolein, crotonaldehyde, 2,4-decadienal, heptadecanal, hexadecenal, 2,4-nonadienal, octadecanal, 2-octenal, pentadecanal, tetradecanal, and trans-2-undecenal were purchased from Tokyo Chemical Industry (Tokyo, Japan). Acetaldehyde, p-toluenesulfonic acid, and the RCs, including propanal, pentanal, butanal, trans-2-hexenal, hexanal, trans-2-heptenal, heptanal, octanal, trans-2-nonenal, nonanal, decanal, undecanal, dodecanal, and tridecanal, were also obtained from Sigma-Aldrich (St. Louis, MO, USA). 4-Hydroxy-2-hexenal (4-HHE) and 4-hydroxy-2-nonenal (4-HNE) were purchased from Cayman Chemical Company (Ann Arbor, MI, USA). p-Benzyloxybenzaldehyde was purchased from Wako Pure Chemical Industries (Osaka, Japan). 8-Heptadecenal, 8,11-heptadecadienal, and 8,11,14-heptadecatrienal were synthesized using a previously described method^50^. Secosterol-A and B were synthesized in accordance with a procedure reported by Wentworth et al.^51,52^. Stock solutions of the RCs were prepared separately in acetonitrile and stored at − 20 °C prior to use.

### Cell culture

HCT116 cells, a human colon adenocarcinoma cell line, were purchased from the American Type Culture Collection (Manassas, VA). HCT116 cells were maintained in Dulbecco’s modified Eagle’s medium supplemented with 10% fetal bovine serum (FBS; HyClone Laboratories Inc., Logan, UT) and antibiotics (100 µg/ml streptomycin and 100 U/ml penicillin, Nacalai Tesque Inc., Kyoto, Japan) at 37 °C with 5% CO_2_. The HCT116 cells were tested and authenticated by JCRB Cell Bank on 21 August 2013.

### Animal experiments

Male C57BL/6-*Apc*^*Min/*+^ mice (Min mice) were purchased from Jackson Laboratory (Bar Harbor, ME, USA). The mice (n = 3–4) were housed in plastic cages with sterilized softwood chips as bedding in a barrier-sustained animal room maintained at 24 ± 2 °C and 55% humidity in a 12-h light/dark cycle. The mice were allowed to drink sterilized water mixed with aspirin at a concentration of 0 or 1000 ppm. Nine or 10 male 5-week-old Min mice were given 0 or 1000 ppm aspirin in drinking water for 8 weeks. Food and water were available ad libitum. The animals were observed daily for clinical symptoms and mortality. Body weight and food consumption were measured weekly. For sacrifice, mice were anesthetized using isoflurane, and blood samples were collected from the abdominal vein. Their intestinal tracts were removed and separated into the small intestine, cecum, and colon. The small intestine was further divided into a proximal segment (4 cm in length) and middle segment and distal segment. The middle and distal segments were each half of the rest of the length after removal of the proximal segment. The number of polyps in the proximal segment was counted at the time of sacrifice with the use of a stereoscopic microscope. The remaining intestinal mucosa (epithelial cells without polyp cells) was removed by scraping, and the specimens were stored at − 80 °C until quantitative real-time PCR analysis. The other regions were opened longitudinally and fixed flat between sheets of filter paper in 10% buffered formalin. Polyp number, size, and intestinal distributions were assessed using a stereoscopic microscope^23^. All experiments were performed in accordance with the “Guidelines for Animal Experiments in the National Cancer Center” and were approved by the Institutional Ethics Review Committee for Animal Experimentation of the National Cancer Center. The animal protocol was designed to minimize pain or discomfort to the animals. The study was reported in accordance with the ARRIVE guidelines.

### Luciferase assays for TCF/LEF, NF-κB, and NRF2 promoter transcriptional activity in stable transfectants

To measure TCF/LEF, NF-κB, and NRF2 transcriptional activities, HCT116 and DLD-1 cells were transfected with 200 ng/well pGL4.49 [luc2P/TCF-LEF/Hygro], pGL4.32 [luc2P/NF-κB/Hygro], and pGL4.37 [luc2P/ARE/Hygro] (Promega, Madison, WI) reporter plasmids using Polyethylenimine HCl MAX, MW 40,000 (Polyscience, Warrington, PA). Cells stably expressing TCF/LEF-, NF-κB-, and NRF2-Luc were treated with hygromycin and cloned. These cells are referred to as HCT116-TCF/LEF-Luc, DLD-1-TCF/LEF-Luc, HCT116-NF-κB-Luc, DLD-1-NF-κB-Luc, HCT116-NRF2-Luc and DLD-1-NRF2-Luc cells, respectively^23^. For the aspirin experiments, the HCT116-TCF/LEF-Luc, DLD-1-TCF/LEF-Luc, HCT116-NF-κB-Luc, DLD-1-NF-κB-Luc, HCT116-NRF2-Luc and DLD-1-NRF2-Luc cells were seeded in 96-well plates (2 × 10^4^ cells per well). After 24 h of pre-incubation, the cells were treated with aspirin for 24 h. Firefly luciferase activity levels were determined using the Bright-Glo Luciferase Assay System (Promega). Basal luciferase activity in the control was set as 1.0. Data are expressed as the mean ± SD (n = 4).

### Quantitative reverse transcription PCR analyses

Total RNA was isolated using RNAiso Plus (Takara Bio, Shiga, Japan) and 1 µg aliquots in a final volume of 20 μL were used for cDNA synthesis using a High-Capacity cDNA Reverse Transcription Kit (Applied Biosystems, Foster City, CA). Real-time PCR was carried out using the CFX96/384 PCR Detection System (Bio-Rad, Tokyo, Japan) and FastStart Universal SYBR Green Mix (Roche Diagnostics, Mannheim, Germany), in accordance with the manufacturer’s instructions^23^. The designed gene primer sequences used to evaluate human and mouse mRNA levels are shown in Supplementary Table 1. To assess the specificity of each primer set used to evaluate human and mouse mRNA levels, the melting curves of the amplicons generated by the PCRs were analyzed.

### Immunohistochemical staining of PCNA to evaluate proliferation of intestinal polyps in min mice

The middle of small intestines were fixed, embedded, and sectioned as Swiss rolls for further immunohistochemical examination using the avidin–biotin complex immunoperoxidase technique after heating with 10 mM citrate buffer (pH 6.0). The primary Ab was monoclonal mouse anti‐PCNA Ab (Calbiochem) at a 100× dilution. As the secondary Ab, biotinylated horse anti‐mouse IgG (Vector Laboratories) was used at a 200× dilution. Staining was undertaken using avidin‐biotin reagents (Vectastain ABC reagents; Vector Laboratories), 3,3’-diaminobenzidine, and hydrogen peroxide, and the sections were counterstained with hematoxylin to facilitate orientation^35^. As a negative control, consecutive sections were immunostained without exposure to the primary Ab. The ratio of PCNA-positive cells was calculated by the formula % = number of PCNA-positive cells per polyp/total number of cells in the polyp (100× magnification).

### Extraction and analysis of reactive carbonyl species

Mouse serum (20 μL) samples were homogenized in 200 μL of sodium phosphate buffer (50 mM, pH 7.4) containing 0.5 mM EDTA and 20 μM BHT. The resulting mixture was agitated vigorously for 1 min and then centrifuged at 15,000 rpm for 10 min, and the organic phase was collected. The remaining precipitate and aqueous phases were then mixed with 400 μL of chloroform/methanol solution (2:1, v/v), and the resulting mixture was centrifuged at 15,000 rpm for 10 min to obtain the organic phase. The combined organic phases were mixed with 100 μL of acetonitrile containing 50 μg of DH and 10 μg of p-toluenesulfonic acid and incubated for 4 h at ambient temperature in the absence of light. The mixtures were then evaporated to dryness in vacuo to yield the corresponding derivatized residues. These residues were dissolved in 200 μL of acetonitrile, and 5 μL of each sample was injected into the LC/MS system. The details regarding RCs analysis by LC/MS were described previously^33,53^.

### Statistical analyses

The results are expressed as mean ± SD and statistical analyses were performed using Student’s *t* test. Differences were considered to be statistically significant at *p < 0.05, **p < 0.01, and ***p < 0.001.

### Supplementary Information

Supplementary Table S1.

Supplementary Figure S1.

## Data Availability

The datasets used and/or analysed during the current study available from the corresponding author on reasonable request.
