# Supplementary material for: Theoretical basis validation and oxidative stress markers for cancer prevention clinical trials of aspirin
Source: Sci Rep. 2023 Dec 11;13:21883. doi: 10.1038/s41598-023-49254-3 (PMC10711014; doi:10.1038/s41598-023-49254-3)
Supplement: Supplementary file 1 — Supplementary Table S1. [file 41598_2023_49254_MOESM1_ESM.docx]

**Supplementary Table 1. Primer sequences**

| **Species** | **Gene name** | **Forward ('5→3')** | **Reverse ('5→3')** |
| --- | --- | --- | --- |
| Human | c-Myc | TCAAGAGGTGCCACGTCTCC | TCTGGCAGCAGGAAGTCCTT |
| Human | Cyclin D1 | CTGTGCTGCGAAGTGGAAACC | GTCCAGGTAGTTCATGGCCAGC |
| Human | IL-6 | ACTCACCTCTTCAGAACGAATTG | GTCGAGGATGTACCGAATTTGT |
| Human | HO-1 | CCAGGCAGAGAATGCTGAGT | GTAGAGAGGGGCGAAGACTG |
| Human | GAPDH | CCACCCATGGCAAATTCC | TGGGATTTCCATTGATGACAA |
| Mouse | c-Myc | GCTCGCCCAAATCCTGTACCT | TCTCCACAGACACCACATCAATTTC |
| Mouse | Cyclin D1 | TGACTGCCGAGAAGTTGTGC | CTCATCCGCCTCTGGCATT |
| Mouse | IL-6 | TGTTCTCTGGGAAATCGTGGA | AAGTGCATCATCGTTGTTCATACA |
| Mouse | HO-1 | GATAGAGCGCAACAAGCAGAA | CAGTGAGGCCCATACCAGAAG |
| Mouse | GAPDH | TTGTCTCCTGCGACTTCA | CACCACCCTGTTGCTGTA |
