# Supplementary material for: Theoretical basis validation and oxidative stress markers for cancer prevention clinical trials of aspirin
Source: Sci Rep. 2023 Dec 11;13:21883. doi: 10.1038/s41598-023-49254-3 (PMC10711014; doi:10.1038/s41598-023-49254-3)
Supplement: Supplementary file 2 — Supplementary Figure S1. [file 41598_2023_49254_MOESM2_ESM.pptx]

## Slide 1
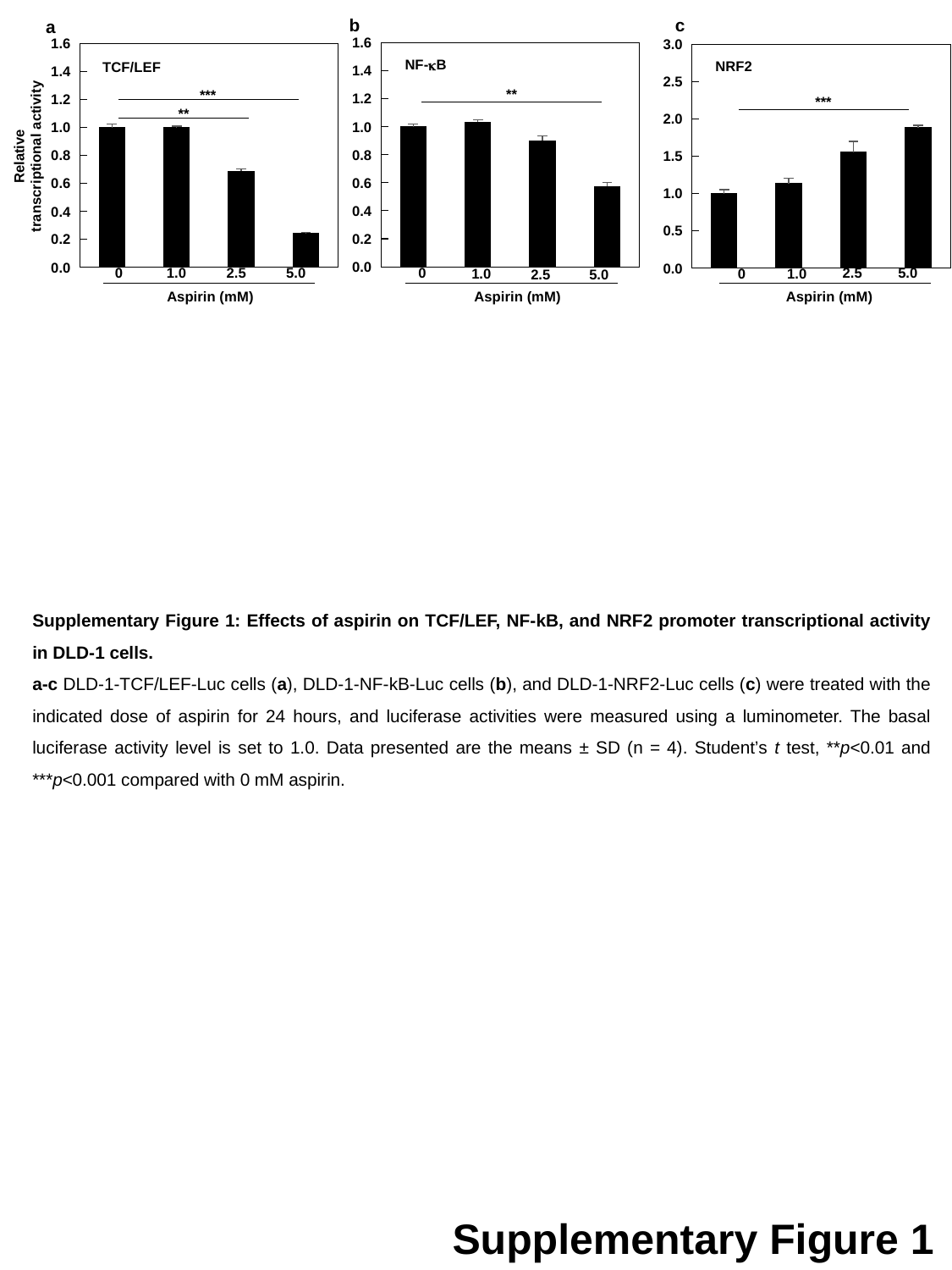

c
b
a
### Chart
| Category | |
|---|---|
| 0 | 1.0 |
| 1 | 1.0331087020216818 |
| 2.5 | 0.9003808965719309 |
| 5 | 0.5727365953706417 |
### Chart
| Category | |
|---|---|
| 0 | 1.0 |
| 1 | 0.9973488411870349 |
| 2.5 | 0.6857949200376293 |
| 5 | 0.24175147524159754 |
### Chart
| Category | |
|---|---|
| 0 | 1.0 |
| 1 | 1.1337031900138697 |
| 2.5 | 1.5547850208044383 |
| 5 | 1.8814147018030514 |NF-kB
NRF2
TCF/LEF
**
***
***
**
Relative
transcriptional activity
0
1.0
2.5
5.0
Aspirin (mM)
5.0
2.5
0
1.0
Aspirin (mM)
0
2.5
5.0
1.0
Aspirin (mM)
Supplementary Figure 1: Effects of aspirin on TCF/LEF, NF-kB, and NRF2 promoter transcriptional activity in DLD-1 cells.
a-c DLD-1-TCF/LEF-Luc cells (a), DLD-1-NF‐kB-Luc cells (b), and DLD-1-NRF2-Luc cells (c) were treated with the indicated dose of aspirin for 24 hours, and luciferase activities were measured using a luminometer. The basal luciferase activity level is set to 1.0. Data presented are the means ± SD (n = 4). Student’s t test, **p<0.01 and ***p<0.001 compared with 0 mM aspirin.
Supplementary Figure 1
